# Primary culture of the rat spinal dorsal horn: a tool to investigate the effects of inflammatory stimulation on the afferent somatosensory system

**DOI:** 10.1007/s00424-020-02478-y

**Published:** 2020-10-24

**Authors:** Stephan Leisengang, Franz Nürnberger, Daniela Ott, Jolanta Murgott, Rüdiger Gerstberger, Christoph Rummel, Joachim Roth

**Affiliations:** 1 Department of Veterinary Physiology and Biochemistry, Justus-Liebig-University Giessen, Frankfurter Strasse 100, 35392 Giessen, Germany; 2 Center for Mind, Brain and Behavior – CMBB, Philipps-University Marburg, Marburg, Germany; 3 Center for Mind, Brain and Behavior – CMBB, Justus-Liebig-University of Giessen, Giessen, Germany

**Keywords:** Somatosensory system, *Substantia gelatinosa*, Primary culture, Inflammation, Neuronal and glial responses

## Abstract

One maladaptive consequence of inflammatory stimulation of the afferent somatosensory system is the manifestation of inflammatory pain. We established and characterized a neuroglial primary culture of the rat superficial dorsal horn (SDH) of the spinal cord to test responses of this structure to neurochemical, somatosensory, or inflammatory stimulation. Primary cultures of the rat SDH consist of neurons (43%), oligodendrocytes (35%), astrocytes (13%), and microglial cells (9%). Neurons of the SDH responded to cooling (7%), heating (18%), glutamate (80%), substance P (43%), prostaglandin E_2_ (8%), and KCl (100%) with transient increases in the intracellular calcium [Ca^2+^]_i_. Short-term stimulation of SDH primary cultures with LPS (10 μg/ml, 2 h) caused increased expression of pro-inflammatory cytokines, inflammatory transcription factors, and inducible enzymes responsible for inflammatory prostaglandin E_2_ synthesis. At the protein level, increased concentrations of tumor necrosis factor-α (TNFα) and interleukin-6 (IL-6) were measured in the supernatants of LPS-stimulated SDH cultures and enhanced TNFα and IL-6 immunoreactivity was observed specifically in microglial cells. LPS-exposed microglial cells further showed increased nuclear immunoreactivity for the inflammatory transcription factors NFκB, NF-IL6, and pCREB, indicative of their activation. The short-term exposure to LPS further caused a reduction in the strength of substance P as opposed to glutamate-evoked Ca^2+^-signals in SDH neurons. However, long-term stimulation with a low dose of LPS (0.01 μg/ml, 24 h) resulted in a significant enhancement of glutamate-induced Ca^2+^ transients in SDH neurons, while substance P-evoked Ca^2+^ signals were not influenced. Our data suggest a critical role for microglial cells in the initiation of inflammatory processes within the SDH of the spinal cord, which are accompanied by a modulation of neuronal responses.

## Introduction

Somatosensory information from peripheral nociceptors and thermosensors is transmitted by afferent axons of dorsal root ganglia (DRG) to neurons in the superficial dorsal horn (SDH) of the spinal cord, including *laminae I* and *II* termed *substantia gelatinosa* [1]. Inflammatory stimulation of the afferent somatosensory system results in an enhanced sensation of pain (hyperalgesia) or sensation of pain to normally non-painful stimuli (allodynia), called inflammatory pain [2]. A number of experimental studies addressed the influence of various mediators of inflammation on the properties and the exaggerated responsiveness of primary nociceptive DRG neurons [3–7]. Enhanced neuronal responses were also reported for dorsal horn neurons of the spinal cord after intrathecal administration of bacterial lipopolysaccharide (LPS) or pro-inflammatory cytokines, which is accompanied by hyperalgesia in vivo [8–11].

Most of the primary afferent neurons are nociceptors or thermoreceptors, especially those with thin myelinated or unmyelinated fibers [12]. These neurons employ glutamate as the principle transmitter to carry noxious or thermal information to the second order neurons located within the SDH [1]. For the synaptic transmission from nociceptive fibers to SDH neurons, also neuropeptides including substance P (SP) were identified [13] and the neurokinin 1 receptor for SP is present in projection neurons in *lamina I* of the spinal cord [14]. Using acutely dissociated dorsal horn cells of neonatal rats, Heath et al. [15] identified a population of neurons, which responded to SP with a short transient increase of intracellular Ca^2+^ concentration ([Ca^2+^]_i_). Ca^2+^ imaging is thus an appropriate tool to characterize the responsiveness of SDH neurons to the relevant transmitters (glutamate and SP) involved in the transmission of nociceptive signals into the spinal cord.

Using a primary culture of rat DRG, we could recently show that glial elements of this structure, satellite glial cells and resident macrophages, play a critical role in the inflammatory enhancement of responses of nociceptive DRG neurons due to stimulation with capsaicin or noxious heat [16]. Therefore, the central goals of the present study were to establish and characterize a mixed neuroglial primary culture of the rat SDH and to investigate whether an enhancement of neuronal responses under conditions of experimentally induced inflammation is also observed in putative second order neurons of the nociceptive pathway. Studies on the effects of transmitters or drugs on the SDH are usually performed with acutely dissociated cells [15] or more refined with spinal cord slice preparations, which preserve the cellular connections within the structure [17, 18]. The mixed neuroglial primary culture of the SDH, which we established for our experiments, gives the opportunity to investigate single cells by means of Ca^2+^ imaging, without being affected by adjacent cells. At the same time, the interplay between all relevant cellular elements of this structure (neurons, astrocytes, microglial cells, and oligodendrocytes) can be investigated. Short- or long-term exposures for hours or even days with inflammatory stimuli can be performed and the effects of inflammatory stimulation can be analyzed at a cellular level not only in neurons, but also in glial cells. With regard to inflammatory and with some extent to neuropathic pain, special attention was directed to spinal cord microglial cells, which were called powerful modulators of pain [19] and which are targets with therapeutic potential [20]. Thus, our primary cell culture model strikes a balance between the complexity of ex vivo slices and simplicity of pure neuronal or glial cell line cultures and is therefore an appropriate tool to study fundamental cellular processes of neuroimmune interactions within the dorsal horn of the spinal cord.

By means of Ca^2+^ imaging, we demonstrate neuronal responses to glutamate, SP, heating, or cooling, which are compatible with data obtained by other experimental approaches. Using lipopolysaccharide (LPS) as a potent inflammatory stimulus, we provide evidence for a critical contribution of glial elements within the SDH primary cultures to the endogenous formation of various mediators of inflammation. We further show that inflammatory stimulation of SDH primary cultures influences stimulus-induced neuronal responses. We suggest that the mixed neuroglial primary culture of the SDH can be a tool to investigate complex neuron–glia interactions at a cellular level and can be used for testing of drugs with the aim to target a modulation of inflammatory pain.

## Material and methods

### Preparation and cultivation of SDH primary cell cultures

Four- to 6-day-old male and female Wistar rats from an in-house breeding colony were used for all experiments. Parent animals originated from Charles River WIGA (Sulzfeld, Germany). Animal care, breeding, and experimental setup were performed according to the guidelines approved by the Hessian Ethics Committee (approval number GI M_580). Environmental conditions were constantly kept at a temperature of 22 °C ± 1 °C and a relative humidity of 50% with lighting from 7:00 AM to 7:00 PM.

For preparation of SDH primary cultures, rat pups were killed by decapitation on days 4 to 6. The vertebral column was removed and cut into slices of about 1 mm. Ten to 15 slices from each animal were collected in Petri dishes filled with cold, oxygenated GBSS (Gey’s Balanced Salt Solution, Sigma-Aldrich Chemie GmbH, Taufkirchen, Germany) supplemented with 0.5% d-glucose (Sigma-Aldrich Chemie GmbH). After removing the vertebral arch, dorsal parts of the spinal cord, including mainly the *laminae I* and *II* (*substantia gelatinosa*), were extracted, cleaned from surrounding spinal meninges, and collected in cold, oxygenated HBSS (Hank’s Balanced Salt Solution, without Ca^2+^ and Mg^2+^; Biochrom GmbH, Berlin, Germany) with 20 mM HEPES (Sigma-Aldrich Chemie GmbH) at pH 7.4. All spinal dorsal horn slices were transferred into an enzyme mix containing dispase II (5 mg/ml; Sigma-Aldrich Chemie GmbH) and collagenase (CLS II, 2.5 mg/ml; Biochrom GmbH) dissolved in oxygenated HBSS. After enzymatic digestion for 40 min, cells were dissociated mechanically and washed in HBSS containing 1 mM EDTA (Sigma-Aldrich Chemie GmbH) to stop the enzymes’ activity. After centrifugation (2 min with 2000 rpm), the supernatant was removed and cells were washed with complete medium, consisting of Neurobasal A supplemented with 2% B27, penicillin (100 U/ml)/streptomycin (0.1 mg/ml) and 2 mM glutamine (All from Life Technologies GmbH, Darmstadt, Germany). Cells were centrifuged, re-suspended in complete medium with a cell number of 100,000 cells/ml, and cultured on poly-l-lysine (0.1 mg/ml; Biochrom GmbH)-coated glass coverslips (Menzel, Braunschweig, Germany). For Ca^2+^ imaging experiments, we used different coverslips with a laser-etched grid (DRM-800 Gridded Coverslips, CELL-VU, Millennium Sciences Inc., New York, USA) for later immunocytochemical identification of all investigated cells. Cultivation was performed in a humidified atmosphere of 5% CO_2_ and 95% air at 37 °C.

### Immunocytochemistry

After ~ 24 h of cultivation, primary cell cultures were fixed with 4% freshly prepared paraformaldehyde (PFA) in phosphate-buffered saline (PBS; both from Sigma-Aldrich Chemie GmbH), pH 7.4, for 20 min. Cells were washed three times in PBS and used for immunocytochemistry.

Immunocytochemistry was performed according the following protocol: After incubation in blocking solution containing 10% fetal calf serum (FCS, Capricorn Scientific GmbH, Ebsdorfer Grund, Germany) diluted in PBS-T containing 0.05% Triton X-100 (Sigma-Aldrich Chemie GmbH) for 2 h, cells were incubated with the primary monoclonal antibodies or polyclonal antisera diluted in blocking solution for 24 h at room temperature in a humidified atmosphere. To remove unbound antibodies, cells were washed three times in PBS-T. Fluorophore-coupled secondary antisera diluted in blocking solution were added for 2 h. After three times washing with PBS-T, cellular nuclei were stained with 2-(4-amidinophenyl)-1H-indole-6-carboxyamidine (DAPI, Life Technologies GmbH) for 8 min. Again, cells were washed three times and coverslips were embedded with a glycerol/PBS solution (Citifluor Ltd., London, UK).

For identification of cell types, we used the following monoclonal antibodies or polyclonal antisera directed against cell type specific marker proteins: MAP2a + b for neurons (mouse AP-20 anti-MAP2a + b; Sigma-Aldrich Chemie GmbH, 1:600), GFAP for astrocytes (mouse anti-GFAP, Merck, Darmstadt, Germany, 1:1000), CNPase for oligodendrocytes (mouse anti-CNPase, Sigma-Aldrich Chemie GmbH, 1:1000), and ED1 for microglial cells (mouse anti-ED1; AbD Serotec, Oxford, UK, 1:1000). Thereafter, cells were incubated with Alexa Fluor 488 donkey anti-mouse IgG (H + L) (Life Technologies GmbH, 1:500). All antibodies have been used previously for cell type detection in several studies [16, 21–23].

**Fig. 1:**
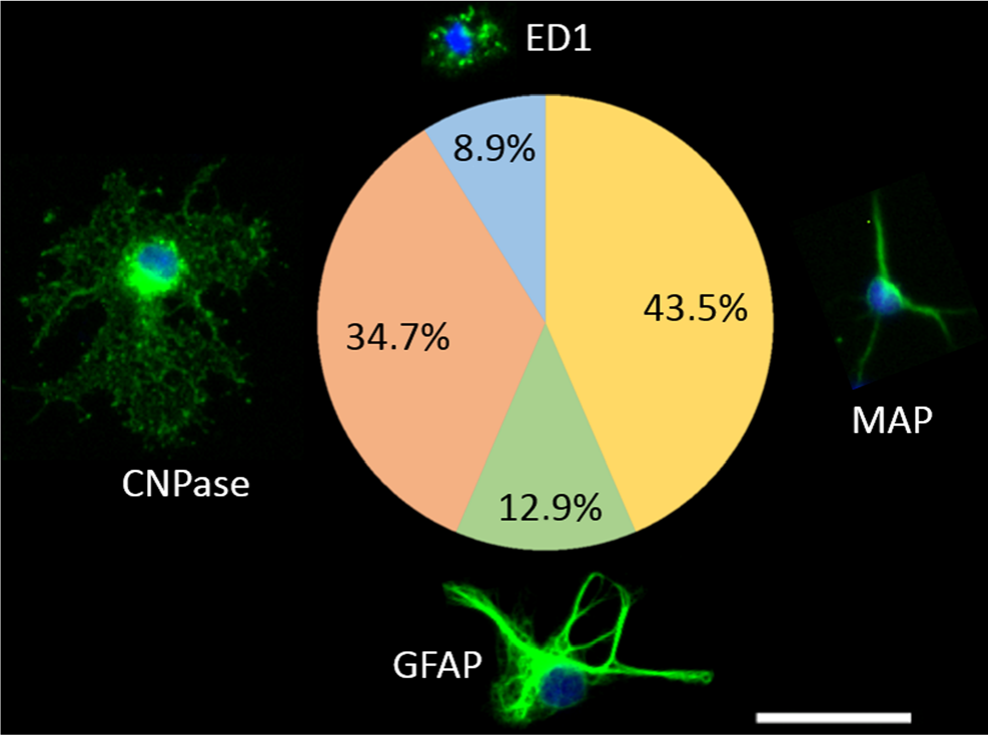
Cell type characterization in SDH primary cultures. Immunocytochemistry was performed to identify neurons (MAP), astrocytes (GFAP), oligodendrocytes (CNPase), and microglial cells (ED1). Percentages represent the number of immunopositive cells of each cell type referring to all investigated immunopositive cells. Scale bar represents 25 μm

The number of immunopositive cells for each cell type was counted in a 1-mm^2^ field on coverslips with a laser-etched grid for six experiments on 21 coverslips with a total number of 1825 cells. Figure 1 shows the percentages of immunopositive cells for each cell-type-specific antibody referring to all investigated immunopositive cells.

**Fig. 2:**
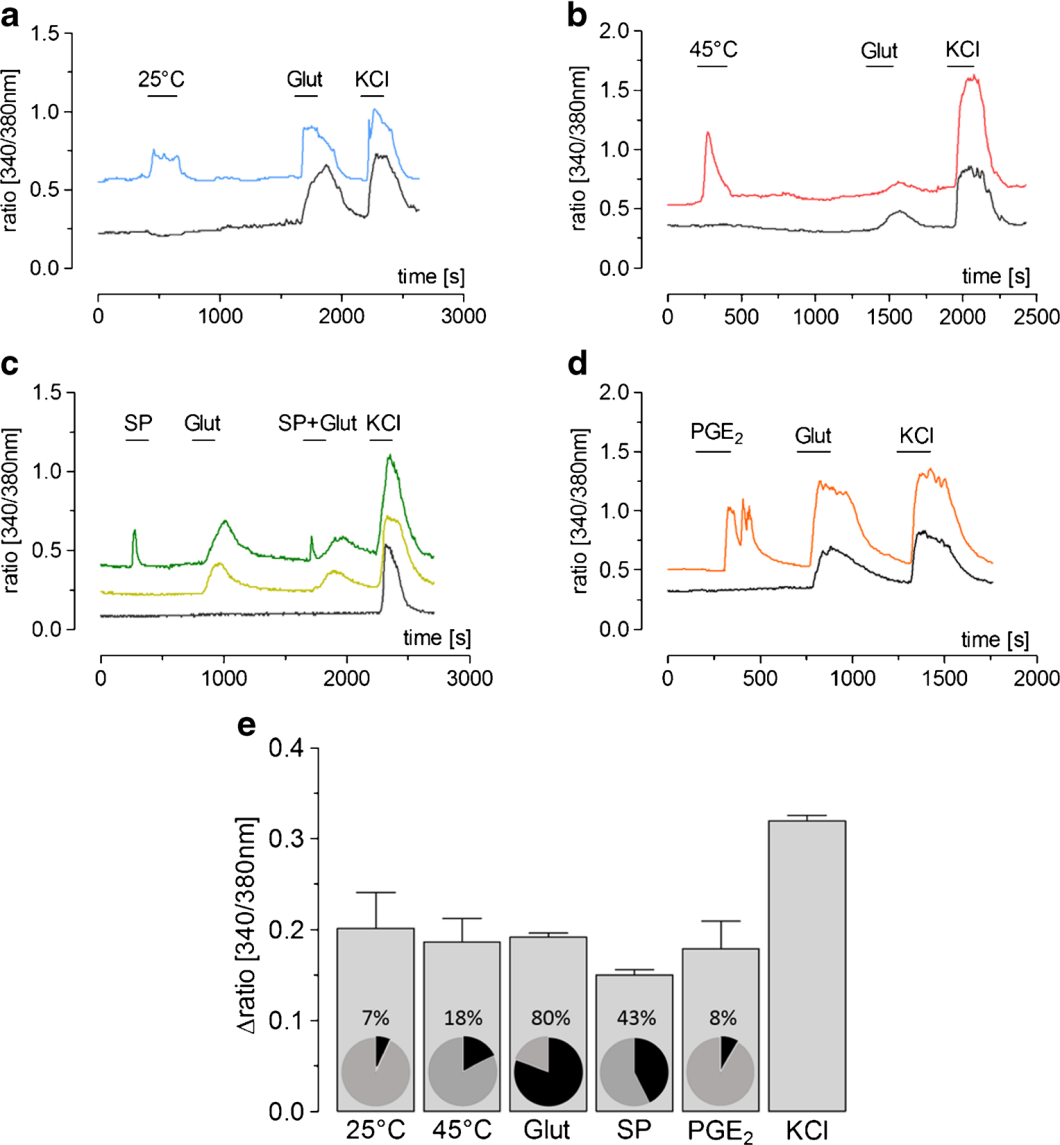
Ca^2+^ responses of SDH neurons to cooling, warming, glutamate, substance P, PGE_2_, and KCl. **a**–**d** Examples of SDH neurons showing direct responses to cooling (**a**), warming (**b**), glutamate (**a**–**d**), substance P (**c**), and PGE_2_ (**d**). KCl was used to test neuronal vitality. **e** Δratio (340/380 nm) values representing mean elevations of [Ca^2+^]_i_ of SDH primary neurons responsive to distinct stimuli and percentages of responsive cells compared to all KCl-responsive vital neurons.

After Ca^2+^ imaging experiments (see Fig. 2), we performed immunocytochemistry for detection of functionally characterized neurons and astrocytes with antibodies/antisera specific for MAP2a + b and GFAP (rabbit anti-GFAP; DAKO GmbH, Hamburg, Germany). Furthermore, the secondary antisera Cy3 goat anti-mouse IgG (H + L) (Dianova GmbH, Hamburg, Germany 1:2000) and Alexa Fluor 488 donkey anti-rabbit IgG (1:500) were applied.

In another experimental setup, we investigated effects of stimulation with LPS on SDH primary cultures by means of immunocytochemistry with regard to cytokine expression and nuclear translocation of inflammatory transcription factors. We therefore used the aforementioned antibodies for cell type specific marker proteins (MAP2a + b, GFAP, CNPase, ED1) in combination with antibodies against the cytokines tumor necrosis factor alpha (TNFα; goat anti-TNFα, R&D Systems, Wiesbaden, Germany; 1:200) and interleukin-6 (IL-6; goat anti-IL-6, Santa Cruz, CA, USA, 1:500) or the transcription factors nuclear factor kappa B (NFκB; rabbit anti-NFκB; Santa Cruz; 1:2000), nuclear factor interleukin-6 (NF-IL6; rabbit anti-NF-IL6, Santa Cruz; 1:4000), phosphorylated cAMP responding element-binding protein (pCREB; rabbit anti-Phospho-CREB (Ser133); Cell Signaling Technology, Danvers, MA, USA; 1:1000), and signal transducer and activator of transcription 3 (STAT3; rabbit anti-STAT3, Santa Cruz; 1:6000). To enhance the signal intensity for cytokine detection, we used a secondary biotinylated antibody (biotinylated horse anti-goat, Vector Laboratories Inc., CA, USA, 1:200 for 2 h) and Cy3-conjugated streptavidin (Jackson ImmunoResearch, Cambridgeshire, UK, 1:1000 for 1 h). For investigation of inflammatory transcription factors, we used the following combination of secondary antisera: Alexa Fluor 488 donkey anti-mouse IgG (1:500) with Cy3-conjugated donkey anti-rabbit IgG (Dianova, Hamburg, Germany; 1:2000).

In all experiments, cells were examined and photographed using a fluorescence microscope (BX-50, Olympus Optical, Hamburg, Germany) equipped with appropriate filter sets and the MetaMorph microscopic imaging software (Molecular Devices, San Jose, USA). To quantify the immunoreactive intensities of a signal in the area of the nucleus, stained with DAPI (blue channel), it was marked as region of interest. The gray level of each pixel in this region was measured in the red channel, corresponding to the signal of the given transcription factor. Results represent the mean of all investigated cells for each cell type from 3 to 4 distinct experiments. In each experiment, PBS- and LPS-treated cells were immunolabeled within the same immunocytochemical procedure and photographed and analyzed under the same conditions.

### Measurement of intracellular calcium

After ~ 24 h of cultivation in complete medium or complete medium containing LPS or PBS, measurements of intracellular calcium were performed. Cells were loaded with 2 μM fura-2-AM (Life Technologies GmbH) in complete medium for 45 min in a humidified atmosphere of 5% CO_2_/95% air at a temperature of 37 °C. Afterwards, coverslips were put in specially constructed Teflon© chambers under an inverted microscope (IMT-2, Olympus GmbH) and superfused with Ca^2+^ imaging buffer consisting of 5 mM HEPES, 130 mM NaCl, 5 mM KCl, 1.0 mM MgCl_2_, 1.25 mM CaCl_2_, and 10 mM d-glucose (all: Sigma-Aldrich Chemie GmbH) at pH 7.4. Temperature and superfusion rate were constantly kept at 37 °C and 2.0 ml/min. Fluorescence measurements were performed using a filter wheel-based excitation system and analyzed with the MetaFluor 7.7.8.0. software (Visitron GmbH, Puchheim, Germany). Regions of interest were defined for single cells and emitted fluorescence (> 515 nm) was detected after alternating excitations at 340 and 380 nm, using a Spot Pursuit digital CCD-camera (Model 23.0, Visitron GmbH) every 5 s during the whole experiment. The 340/380 nm ratios were computed and analyzed. The following stimuli were used in different series of experiments: rapid cooling to 25 °C (240 s), rapid warming to 45 °C (200 s), the TRPV1 agonist capsaicin (1 μM, 180 s), glutamate (10 μM, 180 s) substance P (1 μM, 180 s); all from Sigma-Aldrich Chemie GmbH)), prostaglandin E_2_ (PGE_2_; 10 μM, 180 s; Enzo Life Sciences GmbH, Lörrach, Germany), and buffer containing 50 mM KCl (180 s). All doses were chosen according to previous recordings [16, 24] and pilot studies in which several doses were tested. Drugs were stored as stock solutions at − 20 °C (capsaicin: 10 mM in 0.1% DMSO; glutamate: 100 mM in H_2_O; substance P: 1 mM in 0.6% acetic acid + 1% bovine serum albumin (BSA); PGE_2_: 1 mM in H_2_O) and diluted in Ca^2+^ imaging buffer just prior the experiments. Final solvent concentrations (0.1% DMSO, 0.6% acetic acid + 1% BSA) have been tested in this or previous studies [16] and did not result in changes of [Ca^2+^]_i_.

### LPS stimulation experiments

In one series of experiments, cells were used for short-term stimulation experiments after cultivation in Neurobasal A medium for 24 h. One group was incubated in complete medium containing bacterial lipopolysaccharide (LPS) from *Escherichia coli* O111:B4 (10 μg/ml or 100 μg/ml; Sigma-Aldrich Chemie GmbH, No. L2630) and another group in medium containing solvent (PBS) as a control. After 120 min of incubation in a humidified atmosphere with 5% CO_2_ and 95% air at 37 °C, all supernatants were removed, transferred, and stored at − 45 °C for later determination of cytokines, while the cells were used for Ca^2+^ imaging experiments, immunocytochemistry, or real-time RT-PCR.

In another series of experiments, cells were incubated with complete medium containing LPS (0.001, 0.01, 0.1, or 1 μg/ml) or solvent (PBS) for ~ 24 h in a humidified atmosphere with 5% CO_2_ and 95% air at 37 °C. After this long-term LPS stimulation, supernatants were removed, transferred, and stored at − 45 °C for later determination of cytokines, while cells were used for measurements of intracellular calcium.

### Cytokine measurements

Concentrations of the cytokines TNFα and IL-6 in the supernatants of SDH primary cultures were measured by means of highly sensitive bioassays, which are able to detect even rather low amounts of both cytokines. The TNFα bioassay is based on the cytotoxic effect of TNFα on the mouse fibrosarcoma cell line WEHI 164 subclone 13 [25]. IL-6 was determined by a bioassay based on the dose-dependent growth stimulation of IL-6 on the B9 hybridoma cell line [26]. To calibrate both assays, we used international standards (murine TNFα standard: code 88/532; human IL-6 standard: code 89/548; National Institute for Biological Standards and Control, South Mimms, UK). After considering the dilution of samples into the assays, the detection level for TNFα was 6 pg/ml and for IL-6 3 IU/ml. For detailed description also, see [22, 23].

### Real-time RT-PCR

For determination of gene expression for selected inflammatory target genes, we performed real-time RT-PCR in 4–5 independent experiments. For each experiment, 16 wells were prepared as mentioned before. Eight wells were stimulated with either LPS (10 μg/ml) or PBS for 2 h. After washing with PBS, all cells from 8 wells were lysed in 200 μl RA1 buffer, which is a component of the NucleoSpin© RNA XS kit (Macherey Nagel, Düren, Germany). RNA was extracted according to the manufacturer’s protocol and stored at − 20 °C for later reverse transcription. About 200 ng total RNA were employed for reverse transcription using 50 U of murine leukemia virus reverse transcriptase, 40 μM random hexamers, and 10 μM deoxynucleoside triphosphate (dNTP) mix (Applied Biosystems, Foster City, CA, USA) in a reaction volume of 20 μl. The StepOnePlus Real-Time PCR System (Applied Biosystems) was applied for relative quantification of all probes in duplicates using a primer/probe mixture (TaqMan Gene Expression Assay, Applied Biosystems) and a TaqMan PCR Master Mix (Applied Biosystems) with the following cycling protocol: polymerase activation (50 °C for 2 min), initial denaturation (95 °C for 10 min), 40 cycles of denaturation (95 °C for 15 s), and annealing/elongation (60 °C for 1 min). We used the following gene expression assays from Applied Biosystems: IL-6: Rn01410330_m1; TNFα: Rn99999017_m1; IL-1β: Rn00580432-m1; NF-IL6: Rn00824635_s1; SOCS3: Rn00585674_s1; IκB: Rn01473657_g1; COX-2: Rn01483828_m1; mPGES-1: Rn00572047_m1; Tacr1: Rn00562004_m1.

For normalization of cDNA quantities, we used the housekeeping gene β-actin (Rn00667869_m1; Applied Biosystems) as reference after comparison of different possible housekeeping genes. For relative quantification the 2^−(ΔΔCt)^ method was applied. Results represent the *x*-fold difference in relation to a control sample, which was given a value of 1 within the same experiment for each gene.

### Evaluation and statistics

Ca^2+^ imaging results are presented as mean responses (Δratio [340/380 nm]) ± standard error of the mean (SEM) of all cells that showed an increase of more than 0.05 from their baseline to a distinct stimulus. Percentages within the bars represent numbers of responsive cells in relation to all cells, which were investigated for a given stimulation. In experiments designed to investigate the influence of LPS stimulation on cellular responses, results for each stimulus (substance P or glutamate) were compared by unpaired *t* tests (PBS vs. LPS).

Results from RT-PCR experiments are shown as means ± SEM from 4 to 5 distinct preparations (*n*) and were compared by an unpaired *t* test.

Cytokine concentrations after short-term stimulation with LPS are depicted as means ± SEM from 16 to 19 samples (*n*) from at least 5 distinct experiments and results were analyzed using a *t* test to compare LPS-treated groups with the PBS control group. Results of TNFα measurements after long-term stimulation (~ 24 h) originate from at least 4 distinct experiments and were analyzed using an unpaired *t* test to compare TNFα release of each LPS-treated group to the PBS control group. Columns show the means ± SEM of all investigated samples (*n*).

To compare the mean immunoreactive intensities for different transcription factors within the area of the nucleus, the intensities of 150 to 206 microglial nuclei (*n*) were evaluated as mentioned above and analyzed by an unpaired *t* test. Results originate from 3 to 4 distinct preparations.

The software GraphPad Prism 5 (GraphPad Software Inc., La Jolla, CA, USA) was used for analysis and creation of artwork.

## Results

### Ca^2+^ responses of SDH neurons to thermal and neurochemical stimuli (Fig. 2)

In a first series of experiments, we investigated neuronal responses to thermal and neurochemical stimuli by means of Ca^2+^ imaging after ~ 24 h of cultivation. Changes in the Δratio (340/380 nm), proportional to changes of intracellular calcium ([Ca^2+^]_i_), were analyzed and an increase larger than 0.05 was interpreted as stimulus-induced neuronal response. Neuronal origin of investigated cells was identified by MAP2a + b-specific immunocytochemistry postexperimentally.

Thermal responsiveness of primary SDH neurons was investigated by changing the temperature of Ca^2+^ imaging buffer from 37 °C to either 25 °C or 45 °C. Cooling from 37 to 25 °C resulted in increased [Ca^2+^]_i_ in 25 of 371 investigated neurons (7%) with a mean Δratio (340/380 nm) of 0.2 ± 0.04 (Fig. 2a, e, means ± SEM). Fifty-two of 295 neurons (18%) showed a peak response to warming from 37 to 45 °C (Fig. 2b, e, mean: 0.19 ± 0.03). We further investigated the neuronal responses to glutamate (10 μM, 180 s) and substance P (1 μM, 180 s), two important neurotransmitters within the dorsal horn of the spinal cord involved in the transmission of painful stimuli. About 80% of all investigated neurons (993 of 1234) showed a transient increase of [Ca^2+^]_i_ during stimulation with glutamate (Fig. 2a–e, mean: 0.19 ± 0.005). Two hundred forty-two of 568 neurons (43%) responded to SP with a mean Δratio (340/380 nm) of 0.15 ± 0.006 (Fig. 2c, e). We further looked for a possible effect of co-stimulation with substance P and glutamate at the same time. Co-application of both neurotransmitters resulted in distinct responses with SP inducing a typical fast Ca^2+^ peak and glutamate causing a longer-lasting Ca^2+^ response, which did not overlap with the SP-evoked response (Fig. 2c). Stimulation with prostaglandin E_2_ (PGE_2_; 10 μM, 180 s), a mediator released during spinal inflammation [1, 19], also directly activated a population of neurons cultured from the dorsal horn of the spinal cord. About 8% of all investigated neurons (23 of 271) showed an increase of [Ca^2+^]_i_ due to stimulation with PGE_2_ (Fig. 2d, e, mean: 0.18 ± 0.03). The TRPV1 agonist capsaicin (1 μM, 180 s) did not evoke Ca^2+^ signals in neurons from the SDH (data not shown).

Calcium imaging buffer containing high concentration of KCl (50 mM) was used as a vitality test for neurons at the end of all experiments, leading to a marked elevation of [Ca^2+^]_i_ with mean Δratio (340/380 nm) values of 0.32 ± 0.006 (Fig. 2a–e).

### Inflammatory response of SDH primary cultures to short-term stimulation with LPS (Figs. 3, 4, 5, 6, and 7

**Fig. 3:**
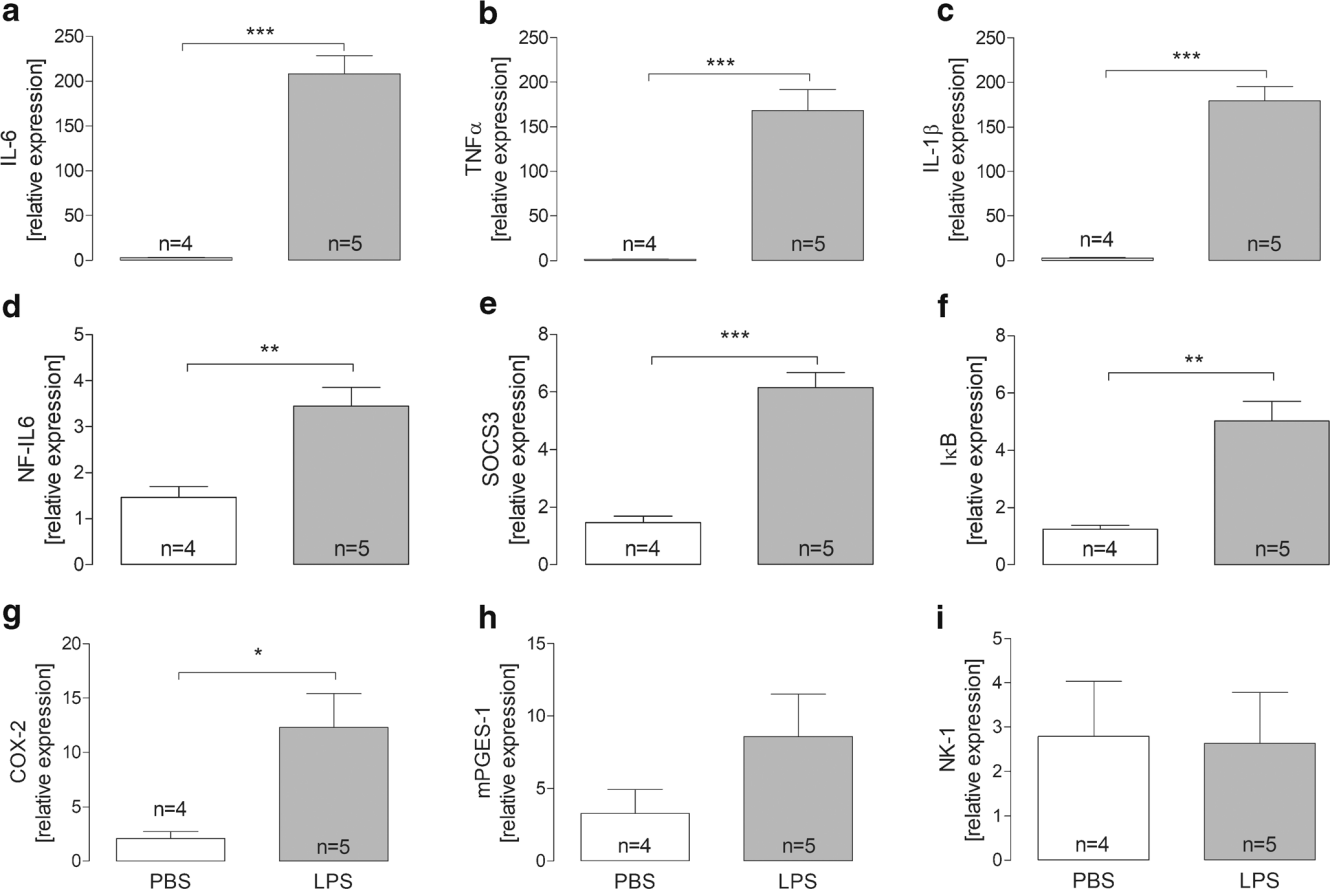
Effects of short-term stimulation with LPS on relative expression of inflammatory marker genes in SDH primary cultures. **a**–**c** After 2 h of stimulation with LPS (10 μg/ml), mRNA expression of pro-inflammatory cytokines (IL-6, TNFα, IL-1β) is significantly increased compared to PBS control groups (***p < 0.001). **d**–**f** Expression of NF-IL6, SOCS3, and IκB is significantly enhanced due to inflammatory stimulation with LPS (**p < 0.01). **g**, **h** COX-2 and mPGES-1 are enzymes involved in the synthesis of PGE_2_. Relative expression of COX-2 is enhanced due to LPS-stimulation (*p < 0.05). **i** Relative expression of the receptor NK-1 was not affected by inflammatory stimulation with LPS for 2 h. Columns represent the means ± SEM of 4 to 5 independent experiments (*n*)

**Fig. 4:**
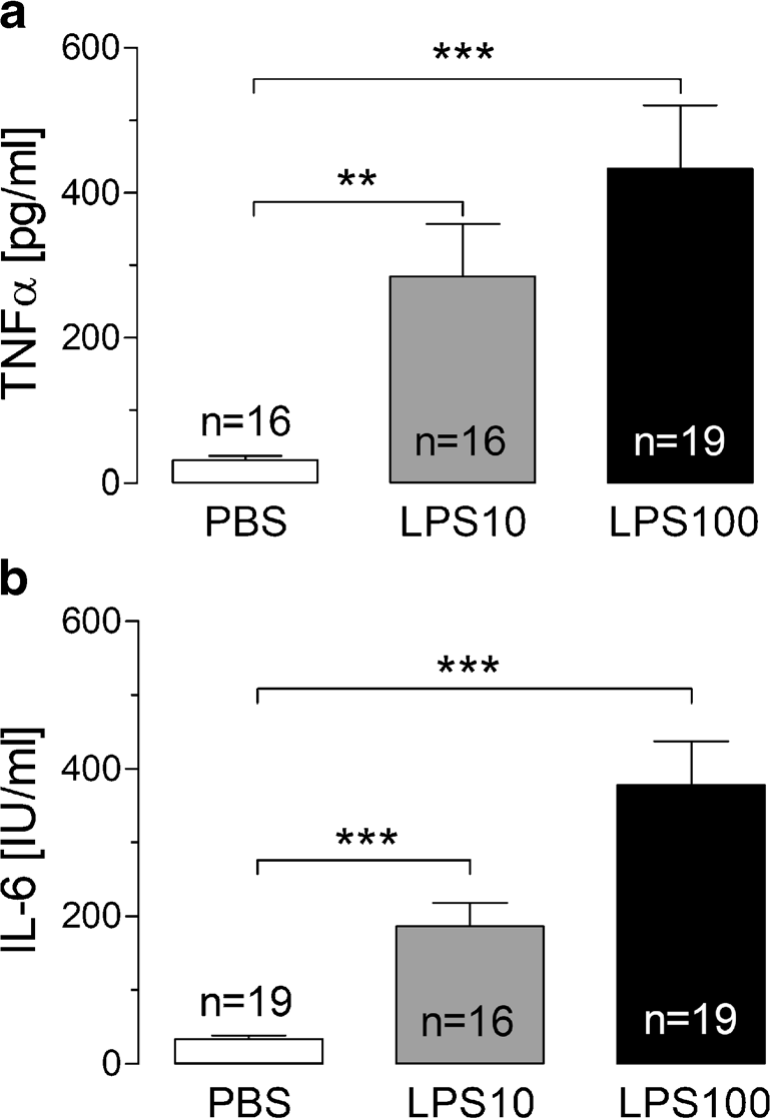
Release of the pro-inflammatory cytokines TNFα and IL-6 into the supernatants of SDH primary cultures after short-term stimulation with LPS. Concentrations of pro-inflammatory cytokines TNFα (**a**) and IL-6 (**b**) are significantly increased after 2 h of stimulation with LPS in different concentrations (10 and 100 μg/ml) compared to the PBS control group (***p < 0.001, **p < 0.01). Columns represent the means ± SEM of 16 to 19 supernatants of SDH primary cultures (*n*) from at least 5 distinct experiments

**Fig. 5:**
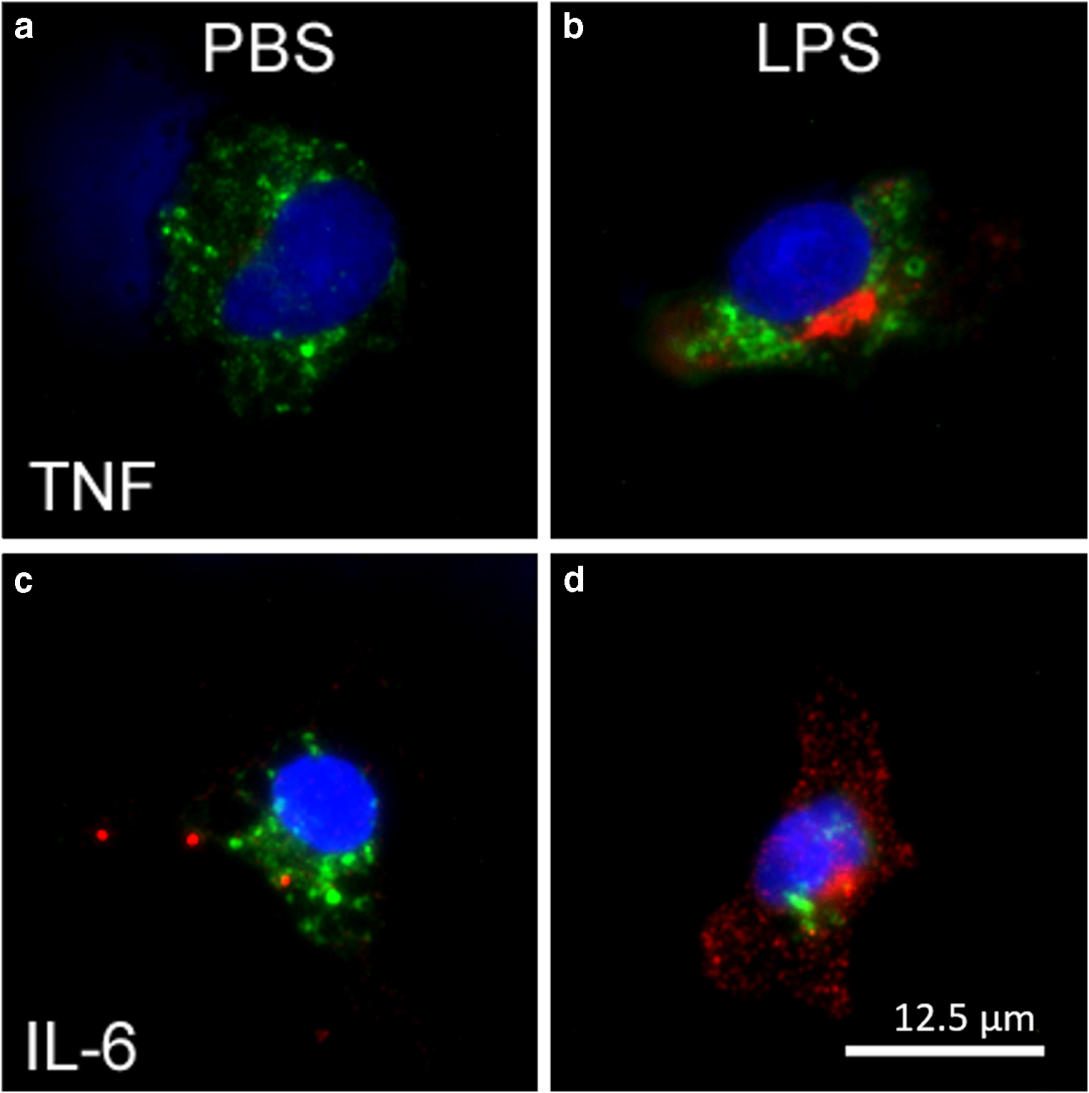
After short-term stimulation with LPS immunoreactivity of TNFα and IL-6 is predominantly induced in SDH microglial cells. **a**, **b** Using antibodies against TNFα (red) and ED1 (green, microglial marker), an increased TNFα-immunoreactivity is detectable in microglial cells after stimulation with LPS (10 μg/ml, 2 h). **c**, **d** LPS stimulation results in increased immunoreactivity for IL-6 (red) in microglial cells (green). Nuclei are stained with DAPI (blue). Scale bar represents 12.5 μm

**Fig. 6:**
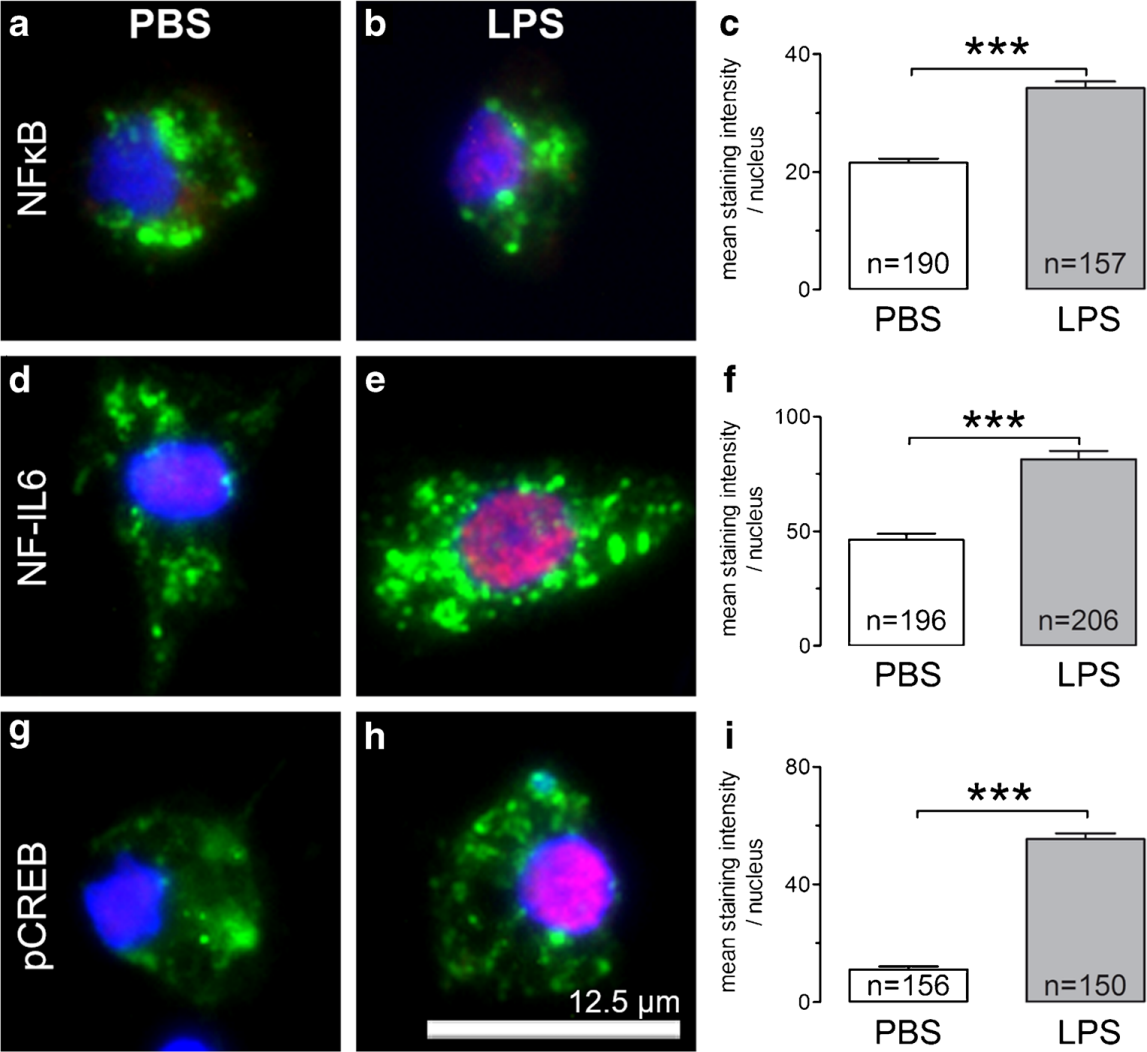
Nuclear translocation of transcription factors NFκB, NF-IL6, and pCREB in microglial cells of SDH primary cultures after short-term stimulation with LPS. Stimulation with LPS (10 μg/ml) for 2 h leads to an increased nuclear immunoreactivity of transcription factors NFκB (**a**, **b** red), NF-IL6 (**d**, **e** red), and pCREB (**g**, **h** red) in microglial cells (ED1, green). Measuring the mean staining intensity within the area of the nucleus (DAPI, blue) in microglial cells a significantly increased immunoreactivity can be observed due to LPS stimulation (**c**, **f**, **i** ***p < 0.001). Columns represent the mean staining intensities ± SEM of all investigated microglial cells (*n*) from at least 3 distinct experiments. Scale bar represents 12.5 μm

**Fig. 7:**
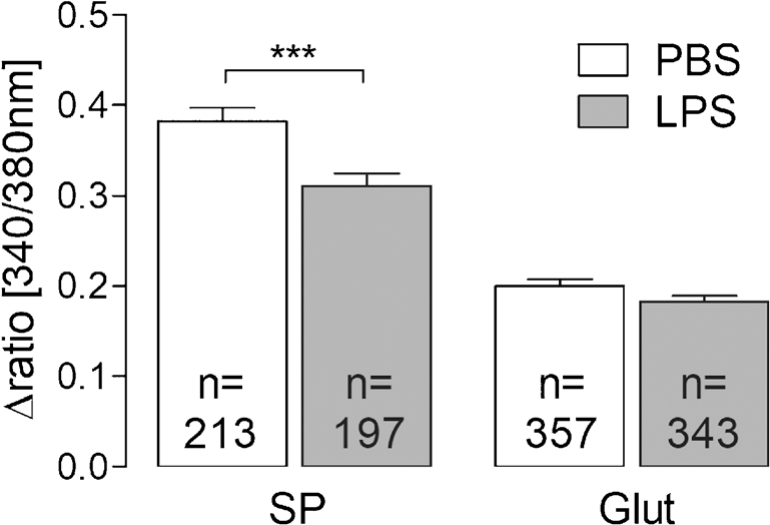
Neuronal Ca^2+^ responses to substance P and glutamate after short-term stimulation with LPS. In Ca^2+^ imaging experiments, the neuronal responses due to stimulation with substance P (1 μM) and glutamate (10 μM) were investigated. Short-term stimulation with LPS (10 μg/ml, 2 h) results in a decreased response to substance P (***p < 0.001), while glutamate responses are not affected significantly (*p* = 0.07). Columns represent the mean stimulus-induced increase of intracellular Ca^2+^ (Δratio [340/380 nm]) ± SEM of all responsive cells (*n*) from 4 distinct preparations

After cultivation for ~ 24 h, we performed a short-term stimulation (2 h) with LPS. To investigate the inflammatory response of SDH primary cultures, RT-PCR (Fig. 3), specific bioassays (Fig. 4), and immunocytochemistry (Figs. 5 and 6) were applied. We further investigated stimulus-induced Ca^2+^ responses after inflammatory stimulation by means of Ca^2+^ imaging (Fig. 7).

After 2 h of stimulation with LPS (10 μg/ml), cultures were washed with PBS. Cells from 8 cultures of each group were lysed and pooled in 200 μl of lysis buffer. RNA was extracted and after reverse transcription, real-time PCR was performed to detect changes in the expression of genes of interest. LPS stimulation led to a significantly increased expression of the pro-inflammatory cytokines TNFα (1.49 ± 0.21 vs. 168.5 ± 23.14, *p* = 0.0004), IL-6 (2.88 ± 0.8 vs. 208.2 ± 20.37, *p* < 0.0001), and IL-1β (2.88 ± 1.0 vs. 179.5 ± 15.85, *p* < 0.0001) (Fig. 3a–c). We further observed an enhanced expression of NF-IL6 (1.47 ± 0.24 vs. 3.44 ± 0.41, *p* = 0.006), SOCS3 (1.47 ± 0.23 vs. 6.16 ± 0.53, *p* = 0.0002), and IκB (1.24 ± 0.13 vs. 5.01 ± 0.7, *p* = 0.002) (Fig. 3d–f). SDH primary cultures also showed an increased expression of COX-2 (2.08 ± 0.65 vs. 12.27 ± 3.14, *p* = 0.026), while no significant changes were detectable for mPGES-1 (3.3 ± 1.63 vs. 8.58 ± 2.95, *p* = 0.19) (Fig. 3g, h). Stimulation with LPS did not affect the expression of NK-1, a receptor for substance P (2.79 ± 1.24 vs. 2.64 ± 1.14) (Fig. 3i).

Supernatants of SDH primary cultures were collected after LPS stimulation (10 or 100 μg/ml). We performed specific bioassays to investigate LPS-induced release of the cytokines TNFα and IL-6. TNFα release was significantly increased after stimulation with 10 μg/ml LPS (284.7 ± 72.54 pg/ml) or 100 μg/ml LPS (433.5 ± 87.63 pg/ml) compared to PBS (31.25 ± 6.14 pg/ml) (Fig. 4a). We further detected a significantly increased release of IL-6 after LPS stimulation (PBS: 33.63 ± 5.45 IU/ml, LPS10: 186.1 ± 32.26 IU/ml, LPS100: 378.2 ± 59.67 IU/ml) (Fig. 4b).

We further investigated effects of short-term stimulation with LPS (10 μg/ml) by means of immunocytochemistry. Using antibodies to detect the cytokines TNFα and IL-6, we were able to identify increased immunoreactivity for both cytokines predominantly in microglial cells (ED1 positive) (Fig. 5), while other investigated cell types (neurons, oligodendrocytes, astrocytes) showed negligible signals (data not shown).

Inflammatory stimuli cause nuclear translocation of transcription factors to activate specific target genes. Accumulation of inflammatory transcription factors within the nuclei of cells can be visualized by means of immunocytochemistry. To determine activation of transcription factors involved in neuroinflammation, we used antibodies for NFκB, NF-IL6, STAT3, and pCREB. NFκB and NF-IL6 were predominantly detectable in microglial cells, while STAT3-immunoreactivity occurred mainly in astrocytes. Nuclear signals of pCREB were observed in microglial cells and in a population of large-sized neurons. Stimulation with LPS (10 μg/ml) for 2 h resulted in increases of nuclear immunoreactivity for NFκB, NF-IL6, and pCREB in microglial cells (Fig. 6). Measuring the mean staining intensities within the area of microglial nuclei (ED1 + DAPI), we were able to show that treatment with LPS results in significantly higher immunoreactivities for NFκB (21.59 ± 0.7 vs. 34.22 ± 1.14, *p* < 0.0001), NF-IL6 (46.39 ± 2.62 vs. 81.39 ± 3.64, *p* < 0.0001), and pCREB (11.17 ± 1.02 vs. 55.43 ± 1.91, *p* < 0.0001) (Fig. 6c, f, i). No significant effects could be detected in other cell types or for STAT3 (data not shown).

To investigate possible effects of short-term stimulation with LPS (10 μg/ml) on neuronal responsiveness, we performed Ca^2+^ imaging experiments. Therefore, substance P (1 μM) and glutamate (10 μM), two important excitatory neurotransmitters within the SDH, responsible for the transmission of pain, were applied as stimuli.

Substance P-evoked Ca^2+^ signals, detectable in about 50% of all investigated neurons, were significantly attenuated after exposure to LPS (10 μg/ml) for 2 h (0.38 ± 0.01; *n* = 213 vs. 0.31 ± 0.01; *n* = 197, *p* = 0.0006). The responses to glutamate in about 90% of all investigated neurons were not affected by LPS stimulation (PBS: 0.2 ± 0.01; *n* = 357 vs. LPS: 0.18 ± 0.01; *n* = 343, *p* = 0.07) (Fig. 7).

### Effects of long-term stimulation with LPS on TNFα release and neuronal Ca^2+^ responses to substance P and glutamate

In order to simulate a long-term low-grade inflammation [16], we cultured SDH primary cell cultures in presence of LPS at different concentrations (0.001, 0.01, 0.1, or 1 μg/ml) for ~ 24 h. Afterwards, supernatants were collected for measurements of TNFα concentrations, while cells were used for Ca^2+^ imaging experiments.

**Fig. 8:**
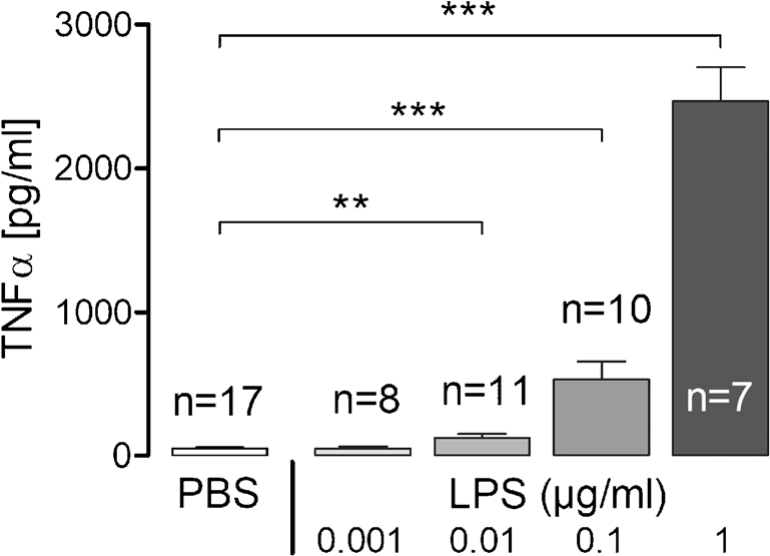
Release of TNFα into supernatants of SDH primary cultures after long-term stimulation with LPS in different concentrations. TNFα release of SDH primary cultures cultivated in presence of LPS (0.01, 0.1, or 1 μg/ml) for ~ 24 h is significantly increased compared to PBS (***p < 0.001, **p < 0.01). The lowest dose of LPS used in this experiment (0.001 μg/ml) does not result in enhanced TNFα concentrations in supernatants. Columns represent the means of *n* investigated primary cultures of at least 4 independent experiments

We tested concentrations from 0.001 to 1 μg/ml to identify the lowest LPS dose showing an effect on TNFα release. A significant increase could be observed in the group treated with 0.01 μg/ml LPS compared to PBS control (123.4 ± 28.66 pg/ml vs. 49.35 ± 6.91 pg/ml, *p* = 0.005), while the lowest dose (0.001 μg/ml) did not significantly affect TNFα release (47.88 ± 15.94 pg/ml, *p* = 0.92). The groups treated with higher LPS doses also showed significantly increased concentrations of TNFα (LPS 0.1: 530.1 ± 127.1 pg/ml, *p* < 0.0001; LPS 1: 2466 ± 234.7 pg/ml, *p* < 0.0001) (Fig. 8). For measurements of intracellular calcium ([Ca^2+^]_i_), we therefore worked with the concentration of 0.01 μg/ml LPS.

**Fig. 9:**
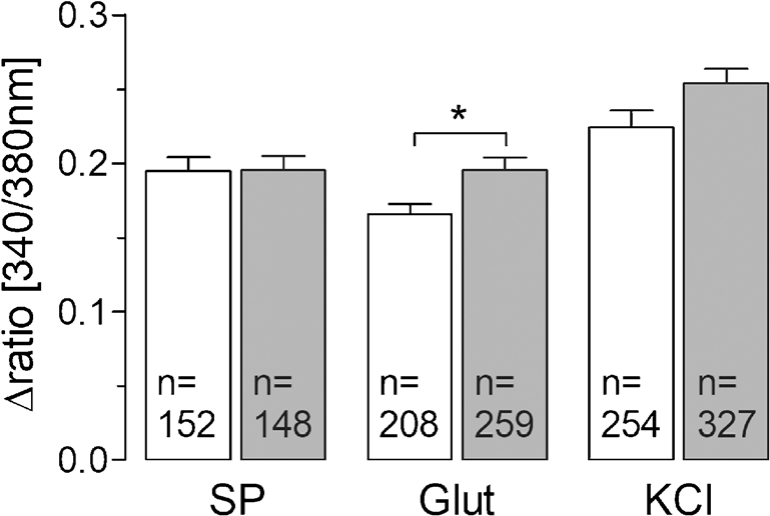
Ca^2+^ responses of neurons from SDH primary cultures after long-term stimulation with LPS. After ~ 24 h of cultivation in presence of LPS (0.01 μg/ml) Ca^2+^ responses of neurons to glutamate are significantly enhanced (***p < 0.001), while there is no effect on substance P responses. *n* represents the number of all responsive neurons to a stimulus from 5 distinct experiments. Columns show the mean increase of [Ca^2+^]_i_ ± SEM of all responsive neurons

After cultivation in presence of LPS (0.01 μg/ml, ~ 24 h), we performed Ca^2+^ imaging experiments to investigate changes in the neuronal responses to the neurotransmitters SP and glutamate. While there was no LPS-induced effect on the Ca^2+^ response of neurons to substance P (0.195 ± 0.01 vs. 0.198 ± 0.012, *p* = 0.83), we observed a significantly increased mean Δratio (340/380 nm) in glutamate-stimulated neurons after long-term LPS pre-treatment (0.166 ± 0.007 vs. 0.211 ± 0.012, *p* = 0.0007) (Fig. 9).

## Discussion

### Neuronal responses of SDH primary cultures to thermal or neurochemical stimulation

In previous studies, we employed mixed neuroglial primary cultures of distinct peripheral [16, 27] and central [21, 22, 28–30] structures of the nervous system. In all these studies, physiological or neurochemical properties of the cultures share some characteristics obtained by other experimental models, like brain slices or in vivo approaches. For example, about 10% of cultured neurons from rat DRG respond to cooling or menthol with a characteristic increase of [Ca^2+^]_i_, thereby suggesting their function as putative cold sensors [16, 31]. Similarly, pronounced Ca^2+^ signals can also be induced by warming primary cultures from the rat preoptic anterior hypothalamus [32] or the median preoptic nucleus (MnPO) [21]. In this study, we aimed to investigate responses of SDH cells to thermal and neurochemical stimuli. Interestingly, cooling or warming of primary cultures from the rat SDH also evoked Ca^2+^ transients in subpopulations of neurons, the number of warm-responsive being substantially higher than the number of cold-responsive neurons (Fig. 2). This observation is in line with classical in vivo studies, showing that selective heating or cooling of the vertebral canal evoked typical thermoregulatory heat or cold defense reactions in anesthetized animals [33, 34]. More than 25 years later, electrophysiological in vitro studies on spinal cord tissue slices provided further evidence for the existence of a population of warm-sensing and a few cold-sensing neurons in the spinal dorsal horn [35]. More recently, ion channels from the transient receptor potential (TRP) family were identified as cellular correlates for neuronal thermoreception. The TRPM8 channel is activated by cold or menthol and was identified as the principle cold sensor in DRG neurons [36]. The TRPM2 channel was characterized as the putative physiological warm sensor in some DRG neurons [37] and in a population of neurons from primary cultures of the preoptic anterior hypothalamus [32]. With regard to thermoreception of neurons within the spinal cord, there is one study suggesting a moderate expression of the TRPM8 cold sensor in the SDH [38]. A neurochemical identification and a functional characterization of the TRPM2 warm sensor within the SDH have to be performed in future studies. Due to the lack of a Ca^2+^ response to stimulation with capsaicin (data not shown), we can exclude the existence of TRPV1 in our culture [39, 40].

Glutamate and SP are important transmitters for the activation of neurons in the SDH by peripheral nociceptors [1, 14]. The distinct kinetics of glutamate- and SP-evoked Ca^2+^ signals allowed to distinguish between the respective responses to both stimuli, even when they were administered at the same time (Fig. 2c). There are further transmitters released within the SDH, which provide excitatory signals for the second-order neurons in the nociceptive pathway including calcitonin gene-related peptide (CGRP) or ATP [13]. The population of neurons within the pain-related pathway in the SDH consists of projection neurons sending ascending axons to the brain and a majority of excitatory and inhibitory interneurons, the axons of which remain in the spinal cord [12, 41]. The primary culture of the SDH, which we employed for our experiments, does not allow to differentiate between projection neurons and interneurons. Still, since many projection neurons possess the neurokinin-1 receptor for SP and are also activated by glutamate [14, 42], we chose these transmitters to analyze a possible impact of experimentally induced inflammation on the responsiveness of SDH neurons (see below).

### Short-term stimulation of SDH primary cultures with a high LPS dose

Intrathecally administered LPS or cytokines produce allodynia and hyperalgesia [9]. We therefore characterized the inflammatory response of mixed neuroglial primary cultures of the SDH to a short-term stimulation for 2 h with an established dose of LPS (10 μg/ml) [16, 28]. The expression of those cytokines (TNFα, IL-1β, and IL-6), which are involved in the inflammation-evoked spinal hyperexcitability [11, 43, 44] and amplification of pain [45], was strongly increased after the LPS exposure (Fig. 3). Corresponding to the cytokine response, we also detected enhanced expression of inflammatory transcription factors. IκB expression indicates activation of NFκB, which plays an important role in the manifestation of hyperalgesia at the level of the spinal cord by upregulation of NFκB-responsive genes including cytokines and COX-2 (Fig. 3: G) [46, 47]. The expression of SOCS3 is indicative of the mobilization of the IL-6-activated transcription factor STAT3. A contribution of this transcription factor to spinal cord inflammation after brain injury was suggested [48] and STAT3 contributes to the formation of PGE_2_ via induction of COX-2 (Fig. 3g) [49]. Specific data for NF-IL6, the third transcription factor showing enhanced LPS-induced expression in SDH cultures (Fig. 3d), are scarce in the context of this study, but its pivotal role in neuroinflammation has been demonstrated in several other studies [50, 51]. However, one study described a potential role for spinal NF-IL6 in the manifestation of neuropathic pain [52]. We failed to demonstrate LPS-induced upregulation of the neurokinin-1 receptor for SP in SDH primary cultures (Fig. 3i). Such an increase seems to occur in vivo 24 h after the injection of Freund’s adjuvant into the hind paw of rats [53]. This discrepancy to our result may be due to stimulus- and time-dependent reasons.

At the protein level, we demonstrated a significant elevation of TNFα and IL-6 in the very small samples of SDH culture supernatants of a few thousand cells (Fig. 4). The production of these cytokines could predominantly be ascribed to microglial cells, which showed increased TNFα and IL-6 immunoreactivities after LPS-stimulation (Fig. 5).

In line with the increased TNFα and IL-6 immunoreactivities in LPS-treated SDH microglial cells, a quantitative evaluation of the nuclear accumulation of inflammatory transcription factors demonstrated the critical role for this cell type in the perception and response to the LPS stimulus (Fig. 6). Increased nuclear immunoreactivity of a given inflammatory transcription factor is indicative of its activation [54]. We demonstrated a LPS-induced increased nuclear translocation of the transcription factors NFκB, NF-IL6, and pCREB in microglial cells of SDH primary cultures (Fig. 6). Activation of NFκB via phosphorylation of IκB by IκB kinase has been shown to be involved in spinal hyperexcitability, especially in the early phase of inflammation, and could be an interesting target for pharmaceutical intervention [46, 47]. The role of NF-IL6 in spinal mechanisms during inflammatory pain has not been investigated in detail so far, but several studies clearly revealed a crucial role of NF-IL6 in neuroinflammatory processes by regulating the expression of inflammatory cytokines TNFα and IL-6 [50, 51, 55, 56]. We also observed an increased nuclear translocation of pCREB in microglial cells of SDH cultures. There is growing evidence that activation of the transcription factor CREB is involved in nociceptive processing [57] and plays a pivotal role in experimentally induced states of inflammatory [58] and neuropathic pain [59–61]. Studies investigating a cell-type-specific activation of CREB in these models promote its function mainly in neurons [59, 62] and also in microglial cells [63, 64]. We also observed nuclear CREB signals in a population of large neurons, but failed to detect a significant LPS-induced effect. Activation of spinal microglial cells in the dorsal horn therefore seems to play a critical role for a subsequent manifestation of neuronal hyperexcitability via release of cytokines and other mediators of inflammation [45, 65, 66]. After 2 h of stimulation with LPS (10 μg/ml), we were not able to show such an effect on SDH neurons in response to substance P or glutamate by means of Ca^2+^ imaging. Instead the short-term inflammatory stimulation resulted in an attenuated SP response. One possible explanation for this finding is an acute release of substance P upon inflammatory stimulation. In studies on sympathetic ganglia, stimulation with LPS or IL-1β resulted in an increased expression of SP only when neurons were co-cultured with non-neuronal cells [67, 68]. Substance P binds to the neurokinin-1 receptor, which is expressed by SDH neurons [14] and activation of NK-1 promptly results in its internalization [69, 70]. A reduced cell surface expression after internalization can be an explanation for our findings of attenuated SP responses due to short-term stimulation with LPS. Further evidence for an internalization of NK-1 could be observed in Ca^2+^ imaging experiments, when SP was applied repeatedly, resulting in a singular SP response in a population of neurons, while others showed reduced SP responses due to the second stimulation (Fig. 2c).

### LPS-induced enhancement of glutamate-evoked Ca^2+^-signals: evidence for glial–neuronal interactions

We aimed to employ the SDH primary culture to establish a correlate for the glial–cytokine–neuronal interactions [65, 66], which finally might lead to enhanced neuronal responses upon inflammatory stimulation as we previously documented for DRG primary cultures [16]. We therefore cultured SDH cells in the presence of a low-dose LPS for ~ 24 h, which was sufficient to evoke an increased release of TNFα into the supernatant (Fig. 8). The long-term stimulation with LPS, indeed, resulted in enhanced neuronal Ca^2+^ signals in response to glutamate, but not to SP (Fig. 9). These data are in line with results obtained by other experimental approaches. For example, previous studies reported that cytokines TNFα, IL-6, or IL-1β released from spinal glial cells caused an increase of glutamatergic synaptic transmission via AMPA or NMDA receptors [8, 45, 65]. A similar effect on NMDA receptors was evoked by interferon-γ [66].

### Possible scenarios for the impact of inflammation on SDH primary cultures

**Fig. 10:**
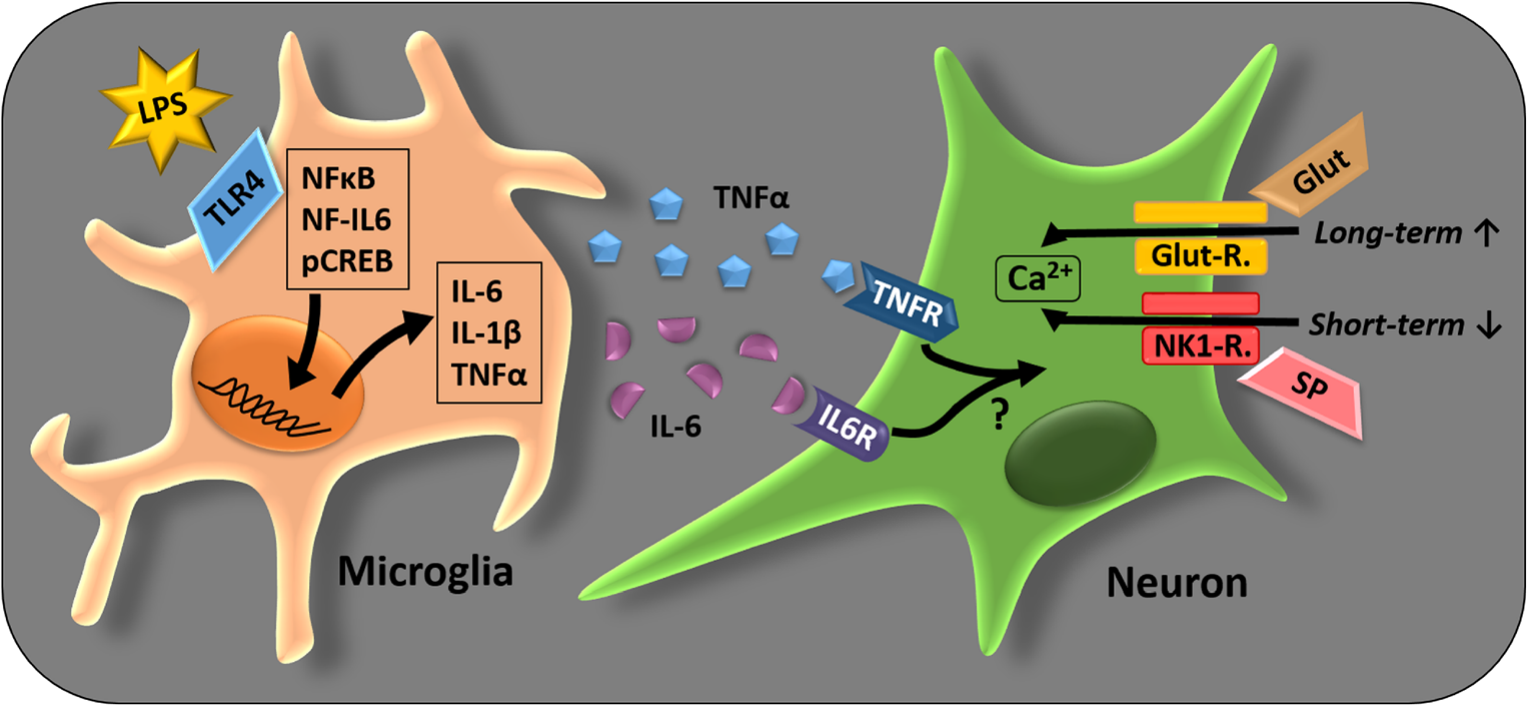
Proposed microglia–neuron interaction in SDH primary cultures. The presented results provide evidence for the following scenario of microglia–neuron interactions in SDH primary cultures: LPS acts on TLR4, expressed by microglial cells, resulting in an increased translocation of transcription factors NFκB, NF-IL6, and pCREB. These transcription factors modulate mRNA expression of pro-inflammatory target genes (e.g., IL-6, IL-1β, TNFα). Inflammatory mediators, like the cytokines TNFα and IL-6, are released into the supernatants. Acting on their receptors on neurons, they are able to modulate the responsiveness to excitatory neurotransmitters. Neurons of SDH primary cultures exposed to a short-term stimulation with LPS (10 μg/ml; 2 h) show reduced Ca^2+^ responses to substance P, while a long-term stimulation for ~ 24 h with a low dose of LPS (0.01 μg/ml) results in increased glutamate responses. Abbreviations: Ca^2+^, calcium; Glut, glutamate; Glut-R., glutamate receptors; IL-1β, interleukin-1 beta; IL-6, interleukin-6; IL6R, interleukin-6 receptor; LPS, lipopolysaccharide; NF-IL6, nuclear factor interleukin-6; NFκB, nuclear factor kappa B; NK1-R., neurokinin 1 receptor; pCREB, phosphorylated cAMP response element-binding protein; SP, substance P; TLR4, Toll-like receptor 4; TNFα, tumor necrosis factor alpha; TNFR, tumor necrosis factor alpha receptors

The two scenarios for putative glial–neural interactions due to short- or long-term stimulation with LPS can therefore be summarized as follows (Fig. 10).

Short-term exposure to a high LPS dose is probably leading to an acute release of substance P, resulting in an internalization of the NK-1 receptor. Long-term stimulation with a low LPS dose, on the other hand, results in sensitization of the glutamate response. The activation of inflammatory transcription factors and release of cytokines from LPS-stimulated microglial cells might build the link to the observed modulation of neuronal responses.

The mixed neuroglial primary culture of the SDH, which we established and introduced in this study, might become an appropriate tool to investigate inhibitors of inflammatory transcription factors [71] or other drugs with proposed capacities to reduce inflammatory pain.

## References

[1] Kuner R (2010). Central mechanisms of pathological pain. Nat Med.

[2] Vega-Avelaira D, Ballesteros JJ, López-García JA (2013). Inflammation-indurced hyperalgesia and spinal microglia reactivity in neonatal rats. Eur J Pain.

[3] Andratsch M, Mair N, Constantin CE, Scherbakov N, Benetti C, Quarta S, Vogl C, Sailer CA, Uceyler N, Brockhaus J, Martini R, Sommer C, Zeilhofer HU, Müller W, Kuner R, Davis JB, Rose-John S, Kress M (2009). A key role of gp130 expressed on peripheral sensory nerves in pathological pain. J Neurosci.

[4] Araldi D, Ferrari LF, Lotufo CM, Vieira AS, Athié MC, Figueiredo JG, Duarte DB, Tambeli CH, Ferreira SH, Parada CA (2013). Peripheral inflammatory hyperalgesia depends on COX increase in the dorsal root ganglion. Proc Natl Acad Sci U S A.

[5] Fang D, Kong LY, Cai J, Li S, Liu XD, Han JS, Xing GG (2015). Interleukin-6-mediated functional upregulation of TRPV1 receptors in dorsal root ganglion neurons through activation of JAK/PI3K signaling pathway: roles in the development of bone cancer pain model. Pain.

[6] Hensellek S, Brell P, Schaible HG, Bräuer R, Segond von Banchet G (2007). The cytokine TNFalpha increases the proportion of DRG neurons expressing the TRPV1 receptor via the TNFR1 receptor and ERK activation. Mol Cell Neurosci.

[7] Russell FA, Veldhoen VE, Tchitchkan D, McDougall JJ (2009). Proteinase-activated receptor-4 (PAR4) activation leads to sensitization of rat joint primary afferents via a bradykinin B2 receptor-dependent mechanism. J Neurophysiol.

[8] Gruber-Schoffnegger D, Drdla-Schutting R, Hönigsperger C, Wunderbaldinger G, Gassner M, Sandkühler J (2013). Induction of thermal hyperalgesia and synaptic long-term potentiation in the spinal cord lamina I by TNF-α and IL-1β is mediated by glial cells. J Neurosci.

[9] Reeve AJ, Patel S, Fox A, Walker K, Urban L (2000). Intrathecally administered endotoxin or cytokines produce allodynia, hyperalgesia and changes in spinal cord neuronal responses to nociceptive stimuli in the rat. Eur J Pain.

[10] Schäfers M, Sorkin L (2008). Effect of cytokines on neuronal excitability. Neurosci Lett.

[11] Zhang L, Berta T, Xu ZZ, Liu T, Park JY, Ji RR (2011). TNF-α contributes to spinal cord synaptic plasticity and inflammatory pain: distinct role of TNF receptor subtypes 1 and 2. Pain.

[12] Todd AJ (2010). Neuronal circuitry for pain processing in the dorsal horn. Nat Rev Neurosci.

[13] Furue H, Katafuchi T, Yoshimura M (2004). Sensory processing and functional reorganization of sensory transmission under pathological conditions in the spinal dorsal horn. Neurosci Res.

[14] Todd AJ, Puskar Z, Spike RC, Hughes C, Watt C, Forrest L (2002). Projection neurons in lamina I of rat spinal cord with the neurokinin 1 receptor are selectively innervated by substance P-containing afferents and respond to noxious stimulation. J Neurosci.

[15] Heath MJS, Womack MD, MacDermott AB (1994). Substance P elevates intracellular calcium in both neurons and glial cells from the dorsal horn of the spinal cord. J Neurophysiol.

[16] Leisengang S, Ott D, Murgott J, Gerstberger R, Rummel C, Roth J (2018). Primary cultures of rat dorsal root ganglia: responses of neurons and glial cells to somatosensory or inflammatory stimulation. Neuroscience.

[17] Biggs JE, Boakye PA, Ganesan N, Stemkowski P, Lantero A, Ballanyi K, Smith PA (2014). Analysis of the long-term actions of gabapentin and pregabalin in dorsal root ganglia and substantia gelatinosa. J Neurophysiol.

[18] Yasaka T, Kato G, Furue H, Rashid MH, Sonohata M, Tamae A, Murata Y, Masuko S, Yoshimura M (2007). Cell-type-specific excitatory and inhibitory circuits involving primary afferents in the substantia gelatinosa of the rat spinal dorsal horn *in vitro*. J Physiol.

[19] Watkins LR, Maier SF (2003). Glia: a novel drug discovery target for clinical pain. Nat Rev Drug Discov.

[20] Inoue K, Tsuda M (2018). Microglia in neuropathic pain: cellular and molecular mechanisms and therapeutic potential. Nat Rev Neurosci.

[21] Leisengang S, Ott D, Gerstberger R, Rummel C, Roth J (2018). Effects of thermal stimulation on neurons and astrocytes cultured from the rat median preoptic nucleus. Neuroreport.

[22] Ott D, Murgott J, Rafalzik S, Wuchert F, Schmalenbeck B, Roth J, Gerstberger R (2010). Neurons and glial cells of the rat organum vasculosum laminae terminalis directly respond to lipopolysaccharide and pyrogenic cytokines. Brain Res.

[23] Simm B, Ott D, Pollatzek E, Murgott J, Gerstberger R, Rummel C, Roth J (2016). Effects of prostaglandin E2 on cells cultured from the rat organum vasculosum laminae terminalis and median preoptic nucleus. Neuroscience.

[24] Ott D, Simm B, Pollatzek E, Gerstberger R, Rummel C, Roth J (2015). Prostaglandin D2 modulates calcium signals induced by prostaglandin E2 in neurons of rat dorsal root ganglia. Neurosci Lett.

[25] Espevik T, Nissen-Meyer J (1986). A highly sensitive cell line, WEHI 164 clone 13, for measuring cytotoxic factor/tumor necrosis factorfrom human monocytes. J Immunol Methods.

[26] Aarden LA, De Groot ER, Schaap OL, Lansdorp PM (1987). Production of hybridoma growth factor by human monocytes. Eur J Immunol.

[27] Rafalzik S, Pehl U, Ott D, Strotmann J, Wolff M, Gerstberger R (2008). Cholinergic signal transduction in the mouse sphenopalatine ganglion. Brain Res.

[28] Grabbe N, Kaspers B, Ott D, Murgott J, Gerstberger R, Roth J (2020). Neurons and astrocytes of the chicken hypothalamus directly respond to lipopolysaccharide and chicken interleukin-6. J Comp Physiol B.

[29] Hatzelmann T, Harden LM, Roth J, Gerstberger R (2013). Antipyretic effect of central [Pyr1]apelin13 on LPS-induced fever in the rat. Regul Pept.

[30] Soares DM, Santos DR, Rummel C, Ott D, Melo MCC, Roth J, Calixto JB, Souza GEP (2017). The relevance of kalikrein-kinin system via activation of B_2_ receptor in LPS-induced fever in rats. Neuropharmacology.

[31] Okazawa M, Takao K, Hori A, Shiraki T, Matsumura K, Kobayashi S (2002). Ionic basis of cold receptors acting as thermostats. J Neurosci.

[32] Song K, Wang H, Kamm GB, Pohle J, Reis FC, Heppenstall P, Wende H, Siemens J (2016). The TRPM2 channel is a hypothalamic heat sensor that limits fever and can drive hypothermia. Science.

[33] Simon E, Iriki M (1971). Ascending neurons highly sensitive to variations of spinal cord temperature. J Physiol.

[34] Simon E (1974). Temperature regulation: the spinal cord as a site of extrahypothalamic thermoregulatory functions. Rev Physiol Biochem Pharmacol.

[35] Pehl U, Simon E, Schmid HA (1997). Properties of spinal neuronal thermosensitivity in vivo and in vitro. Ann N Y Acad Sci.

[36] Bautista DM, Siemens J, Glazer JM, Tsuruda PR, Basbaum AI, Stucky CL, Jordt SE, Julius D (2007). The menthol receptor TRPM8 is the principal detector of environmental cold. Nature.

[37] Tan CH, McNaughton PA (2016). The TRPM2 ion channel is required for sensitivity to warmth. Nature.

[38] Kim YS, Park JH, Choi SJ, Bae JY, Ahn DK, McKemy DD, Bae YC (2014). Central connectivity of transient receptor potential melastatin 8-expressing axons in the brain stem and spinal dorsal horn. Public Library of Science One.

[39] Garami A, Shimansky YP, Pakai E, Oliveira DL, Gavva NR, Romanovsky AA (2010). Contributions of different modes of TRPV1 activation to TRPV1 antagonist-induced hyperthermia. J Neurosci.

[40] Romanovsky AA, Almeida MC, Garami A, Steiner AA, Norman MH, Morrison SF, Nakamura K, Burmeister JJ, Nucci TB (2009). The transient receptor potential vanilloid-1 channel in thermoregulation: a thermosensor it is not. Pharmacol Rev.

[41] Todd AJ (2017). Identifying functional populations among the interneurons in laminae I-III of the spinal dorsal horn. Mol Pain.

[42] Polgár E, Al Ghamdi KS, Todd AJ (2010). Two populations of neurokinin 1 receptor-expressing projection neurons in lamina I of the rat spinal cord that differ in AMPA receptor subunit composition and density of excitatory synaptic input. Neuroscience.

[43] König C, Morch E, Eitner A, Möller C, Turnquist B, Schaible HG, Ebersberger A (2016). Involvement of spinal IL-6 *trans*-signaling in the induction of hyperexcitability of deep dorsal horn neurons by spinal tumor necrosis factor-alpha. J Neurosci.

[44] Vazquez E, Kahlenbach J, Segond von Banchet G, König C, Schaible HG, Ebersberger A (2012). Spinal interleukin-6 is an amplifier of arthritic pain in the rat. Arthritis Rheum.

[45] Kawasaki Y, Zhang L, Cheng JK, Ji RR (2008). Cytokine mechanisms of central sensitization: distinct and overlapping role of interleukin-1β, interleukin-6, and tumor necrosis factor-α in regulating synaptic and neuronal activity in the superficial spinal cord. J Neurosci.

[46] Ebersberger A, Buchmann M, Ritzeler O, Michaelis M, Schaible HG (2006). The role of spinal nuclear factor-kappa B in spinal hyperexcitability. Neuroreport.

[47] Tegeder I, Niederberger E, Schmidt R, Kunz S, Gühring H, Ritzeler O, Michaelis M, Geisslinger G (2004). Specific inhibition of IκB kinase reduces hyperalgesia in inflammatory and neuropathic pain models in rats. J Neurosci.

[48] Dominguez E, Mauborgne A, Mallet J, Desclaux M, Pohl M (2010). SOCS3-mediated blockade of JAK/STAT3 signaling pathway reveals its major contribution to spinal cord neuroinflammation and mechanical allodynia after peripheral nerve injury. J Neurosci.

[49] Rummel C, Sachot C, Poole S, Luheshi GN (2006). Circulating interleukin-6 induces fever through a STAT3-linked activation of COX-2 in the brain. Am J Physiol Regul Integr Comp Physiol.

[50] Fuchs F, Damm J, Gerstberger R, Roth J, Rummel C (2013). Activation of the inflammatory transcription factor nuclear factor interleukin-6 during inflammatory and psychological stress in the brain. J Neuroinflammation.

[51] Schneiders J, Fuchs F, Damm J, Herden C, Gerstberger R, Soares DM, Roth J, Rummel C (2015). The transcription factor nuclear factor interleukin 6 mediates pro- and anti-inflammatory responses during LPS-induced systemic inflammation in mice. Brain Behav Immun.

[52] Yi H, Liu S, Kashiwagi Y, Ikegami D, Huang W, Kanda H, Iida T, Liu CH, Takahashi K, Lubarsky DA, Hao S (2018). Phosphorylated CCAAT/enhancer binding protein β contributes to rat HIV-related neuropathic pain: *in vitro* and *in vivo* studies. J Neurosci.

[53] Duric V, McCarson K (2007). Neurokinin-I (NK-I) receptor and brain-derived neurotrophic factor (BDNF) gene expression is differentially modulated in the rat spinal dorsal horn and hippocampus during inflammatory pain. Mol Pain.

[54] Rummel C (2016). Inflammatory transcription factors as activation markers and functional readouts in immune-to-brain communication. Brain Behav Immun.

[55] Akira S, Isshiki H, Sugita T, Tanabe O, Kinoshita S, Nishio Y, Nakajima T, Hirano T, Kishimoto T (1990). A nuclear factor for IL-6 expression (NF-IL6) is a member of a C/EBP family. EMBO J.

[56] Pope RM, Leutz A, Ness SA (1994). C/EBP beta regulation of the tumor necrosis factor alpha gene. J Clin Investig.

[57] Niederberger E, Ehnert C, Gao W, Coste O, Schmidtko A, Popp L, von Gall C, Korf HW, Tegeder I, Geisslinger G (2007). The impact of CREB and its phosphorylation at Ser142 on inflammatory nociception. Biochem Biophys Res Commun.

[58] Ji RR, Rupp F (1997). Phosphorylation of transcription factor CREB in rat spinal cord after formalin-induced hyperalgesia: relationship to c-fos induction. J Neurosci.

[59] Crown ED, Ye Z, Johnson KM, Xu G-Y, McAdoo DJ, Westlund KN, Hulsebosch CE (2005). Upregulation of the phosphorylation form of CREB in spinothalamic tract cells following spinal cord injury: relation to central neuropathic pain. Neurosci Lett.

[60] Ma W, Quirion R (2001). Increased phosphorylation of cyclic AMP response element-binding protein (CREB) in the superficial dorsal horn neurons following partial sciatic nerve ligation. Pain.

[61] Seybold VS, McCarson KE, Mermelstein PG, Groth RD, Abrahams LG (2003). Calcitonin gene-related peptide regulates expression of neurokinin_1_ receptors by rat spinal neurons. J Neurosci.

[62] Lonze BE, Ginty DD (2002). Function and regulation of CREB family transcription factors in the nervous system. Neuron.

[63] Herdegen T, Fiallos-Estrada C, Schmid W, Bravo R, Zimmermann M (1992). The transcription factor CREB, but not immediate-early gene encoded proteins, is expressed in activated microglia of lumbar spinal cord following sciatic nerve transection in the rat. Neurosci Lett.

[64] Kim H, Moon C, Ahn M, Lee Y, Kim S, Matsumoto Y, Koh C-S, Kim M-D, Shin T (2007). Increased phosphorylation of cyclic AMP response element-binding protein in the spinal cord of Lewis rats with experimental autoimmune encephalomyelitis. Brain Res.

[65] Guo W, Wang H, Watanabe M, Shimizu K, Zou S, LaGraize SC, Wie F, Dubner R, Ren K (2007). Glial-cytokine-neuronal interactions underlying the mechanisms of persistent pain. J Neurosci.

[66] Sonekatsu M, Taniguchi W, Yumanaka M, Nishio N, Tsutsui S, Yamada H, Yoshida M, Nakatsuka T (2016). Interferon-gamma potentiates NMDA receptor signaling in spinal dorsal horn neurons via microglia-neuron interaction. Mol Pain.

[67] Freidin M, Kessler JA (1991). Cytokine regulation of substance P expression in sympathetic neurons. Proceedings of the National Academy of Sciences USA.

[68] Shadiack AM, Carlson CD, Ding M, Hart RP, Miller Jonakait G (1993). Lipopolysaccharide induces substance P in sympathetic ganglia via ganglionic interleukin-1 production. J Neuroimmunol.

[69] Mantyh PW, DeMaster E, Malhotra A, Ghilardi JR, Rogers SD, Manty CR, Liu H, Basbaum AI, Vigna SR, Maggio JE, Simone DA (1995). Receptor endocytosis and dendrite reshaping in spinal neurons after somatosensory stimulation. Science.

[70] Marvizón JCG, Wang X, Matsuka Y, Neubert JK, Spigelman I (2003). Relationship between capsaicin-evoked substance P release and neurokinin 1 receptor internalization in the rat spinal cord. Neuroscience.

[71] Zhao D, Kwon SH, Chun YS, Gu MY, Yang HO (2017). Anti-neuroinflammatory effects of fucoxanthin via inhibition of Akt/NF-κB and MAPKs/AP-1 pathways and activation of PKA/CREB pathway in lipopolysaccharide-activated BV-2 microglial cells. Neurochem Res.

